# Evaluation of two laboratory model methods for diarrheal irritable bowel syndrome

**DOI:** 10.1186/s10020-022-00599-x

**Published:** 2023-01-12

**Authors:** Qian Chen, Hua Zhang, Chang-Yue Sun, Qing-Ying He, Rui-Rong Zhang, Bin-Fei Luo, Zi-Hao Zhou, Xiao-Fan Chen

**Affiliations:** 1 Evidence-Based Medicine Research Centre, Jiangxi University of Chinese Medicine, Nanchang, 330004 Jiangxi China; 2 Department of Food Nutrition and Safety, College of Pharmacy, Jiangxi University of Chinese Medicine, Nanchang, 330004 Jiangxi China; 3 Chengdu University of Traditional Chinese Medicine, Chengdu, 611137 China

**Keywords:** Diarrheal irritable bowel syndrome, Animal model, Stress, Gut microbiota

## Abstract

**Background:**

Diarrheal irritable bowel syndrome (IBS-D) is a common chronic functional gastrointestinal disorder, and the underlying pathogenic mechanism is still unclear. Animal models that mimic the pathological state of IBS-D patients were constructed to provide a reference for later drug research and model development.

**Methods:**

The IBS-D model was induced using restraint stress and chemical stimulation (rhubarb), and rats were divided into normal control group (NC), chemically stimulated group (CS), and restraint stress group (RS). Visceral motility responses to Colorectal Balloon Dilation (CRD) were measured by Abdominal Withdrawal Reflex (AWR); evaluation of faecal properties and water content; determination of colonic tissue tight junction (TJ) mRNA expression by RT-PCR; measurement of inflammatory cytokines by ELISA; and intestinal flora and short chain fatty acids.

**Results:**

Compared to NC group, CS and RS group rats showed increased intestinal sensitivity and Bristol stool score, significant diarrheal symptoms and weight loss. Mucin 2, ZO-1, OCLN, CLDN4 mRNA expression was reduced and the intestinal mucosal barrier function was diminished. In addition, the levels of inflammatory factors IL-1β, IL-6, IL-8, IL-10 and TNF-α increased, the abundance and diversity of intestinal flora decreased, the content of beneficial bacteria such as *Bifidobacteria* decreased, and SCFAs such as acetic acid, propionic acid and butyric acid decreased to different degrees. Although, no significant difference was observed for any molecular and inflammatory marker, but compared to CS group, RS group had less water in the stool, higher visceral sensitivity, and higher relative abundance of beneficial intestinal bacteria such as *Actinobacteria*.

**Conclusion:**

In conclusion, restraint stress combined with chemical stimulation can mimic the pathological state of diarrhoea symptoms, visceral hypersensitivity, reduced intestinal mucosal barrier permeability, immune regulatory dysfunction and dysbiosis in IBS-D patients. However, herbs with antibacterial effects such as rhubarb and senna, for example, are not suitable as the first choice for chemical stimulation, as they may lead to a decrease in harmful bacteria and an increase in beneficial bacteria in the intestinal fraction and do not perfectly mimic the imbalanced state of intestinal flora in IBS-D patients, while restraint stress may be a key factor in modelling.

**Supplementary Information:**

The online version contains supplementary material available at 10.1186/s10020-022-00599-x.

## Background

Irritable bowel syndrome (IBS) is a common chronic functional gastrointestinal disorder characterized by recurrent abdominal pain or discomfort associated with abnormal bowel habits, and epidemiological data show that the prevalence of IBS varies widely worldwide, with the prevalence of irritable bowel syndrome being approximately 12% in North America and IBS being most prevalent in South America (21.0%) and least prevalent in Southeast Asia (7.0%) (Chey et al. 2015; Lacy et al. 2021; Black and Ford 2020). Diarrheal irritable bowel syndrome (IBS-D) is the main subtype of IBS, which mainly manifests clinically as abdominal pain and diarrhea and seriously affects the quality of life of individuals and brings a certain economic burden (Drossman 2016; Lovell and Ford 2012; Fukudo et al. 2021).

The pathogenesis of IBS-D cannot be explained by structural or biochemical abnormalities, and current causative factors include food intolerance, stress, altered flora, visceral hypersensitivity, immune dysregulation, gut-brain axis dysregulation, and genetic factors, but the underlying mechanisms are unclear (Sebastián Domingo 2022). Among them, food intolerance and stress are two very common causes (Scuderi et al. 2020; Radovanovic-Dinic et al. 2018). Animal models that mimic disease pathogenesis and symptoms are essential for the research of disease, which may help in the development of new therapeutic approaches. The methods used to produce animal models of IBS-D in reported studies include restraint stress, water avoidance stress, neonatal maternal separation, chemical stimulation, and mechanical stimulation (Chong et al. 2019). Although most animal models have altered visceral hypersensitivity similar to that of IBS, there is a need to establish accurate and valid criteria to evaluate the objectivity and validity of the models. The existing animal models of IBS-D have their own advantages and disadvantages and need to be continuously improved (Vannucchi and Evangelista 2018; Enqi et al. 2020; Qin et al. 2011).

Restraint stress and chemical stimulation is one of the most common modelling methods for IBS-D. In order to evaluate the effectiveness of the restraint stress combined with chemical stimulation model, we conducted a 28-day study using a rat model to examine intestinal sensitivity, faecal properties, intestinal histopathological changes, inflammatory cytokines, intestinal flora and short-chain fatty acids (SCFAs) to further understand the characteristics of the animal model of IBS-D and to provide a reference for establishing a better model for drug research and development.

## Materials and methods

### Materials and reagents

Rhubarb was purchased from Jiangxi Jiangzhong TCM Decoction Pieces Co., LTD. (200,709), IL-1β, IL-6, IL-8, IL-10, TNF-α ELISA kit was purchased from Boster Bioengineering Co., LTD., IgA was purchased from Neobioscience. Antibodies zonulae occluden-1 (ZO-1) and mucin 2 (Muc2) were purchased from Boster Bioengineering Co., LTD. (PB9234, BM5029).

### Animals

Thirty SPF SD male rats, body mass (180 ± 20) g, purchased from Jiangxi University of Chinese Medicine, Animal Certificate No. SCXK (Gan) 2018–0003, were housed at (23 ± 2)°C in half light and half dark each day. Rats were fed sterile standard chow, purchased from Jiangxi University of Traditional Chinese Medicine, and fed ad libitum with food and water for 1 week after acclimatization for use. The rats were divided into 3 groups of 10 rats each: healthy control group (NC), chemically stimulated group (CS), and restraint stress group (RS). The study was reviewed by the Experimental Animal Ethics Committee of Jiangxi University of Chinese Medicine.

### IBS-D animal model establishment

**Fig. 1:**
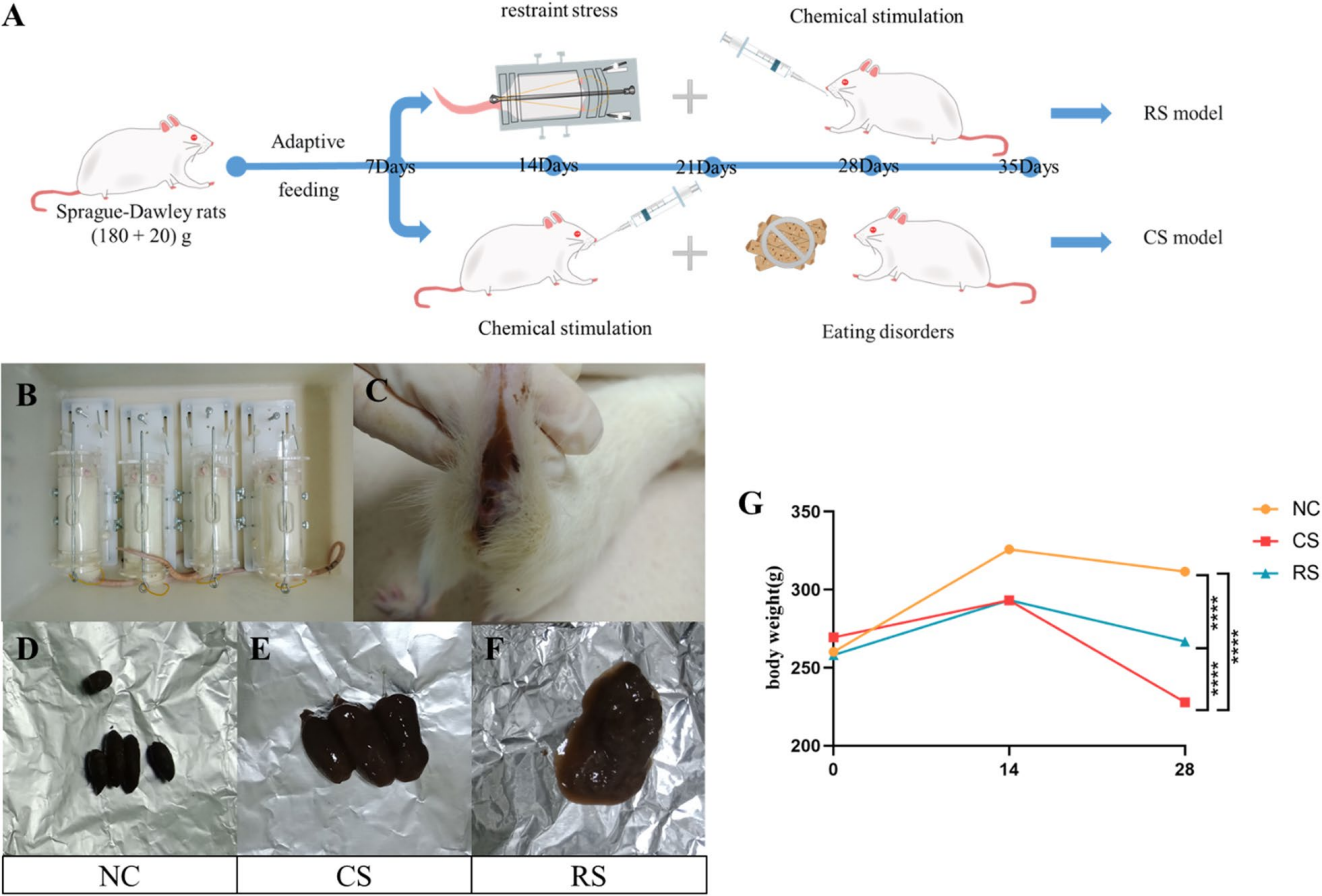
The detailed procedures for rat experiments in five models and their general conditions. **A** Experimental steps for both models. **B** Restraint during moulding; **C** perianal obscuration of rats after moulding; **D** faeces of rats in NC group; **E** faeces of rats in CS group; **F** faeces of rats in RS; **G** changes in body weight of rats. ****p < 0.0001

After 1 week of adaptive feeding, daily gavage of rhubarb aqueous decoction combined with restraint stress for 4 weeks was used to establish a restraint stress IBS-D model (RS) (Zhu et al. 2019). A chemically stimulated IBS-D model (CS) was established by controlling the diet (one day of satiety and one day of fasting without water restriction) combined with daily gavage of aqueous decoction of rhubarb for 4 weeks (Fig. 1A). After successful modeling, the abdominal wall retreat test score (AWR) was detected using the colorectal dilatation (CRD) method, and the Bristol stool score and stool water content were tested. Among them, AWR score of 2 or above and stool score of 5 or above represented successful model establishment.

### Measurement of AWR scores

The rats were fasted for 12 h before the experiment, anesthetized with ether, and the balloon coated with paraffin oil was inserted into the colorectum so that the end of the balloon penetrated 1.0 cm into the anus, and the catheter was fixed at 1.0 cm outside the anus with adhesive tape at the root of the rat's tail. The rats were placed in a 20 cm × 6 cm × 8 cm Plexiglas observation box, and the experiment was started after 30 min of complete adaptation. CRD pressures of 20, 40, 60, and 80 mmHg were used, and each dilatation lasted 20 s with a stimulation interval of 4 min, and the average of the three scores was taken. The pressure changes were observed and recorded by the person who injected the gas, and the behavioral responses of the rats were observed by another person during the gas injection, and the strength of the abdominal wall retreat reflex was scored, and the minimum pressure value at which the lower abdominal wall was visually observed to lift off the bottom of the box or to flatten with obvious contraction (AWR score of 3) was used as the pain threshold, and the dilatation pressure range was 0 ~ 80 mmHg. AWR scoring criteria are shown in Table 1 (Lin et al. 2007).

**Table 1:** AWR scoring standards

Points	Behavioral changes in rats
0	No significant behavioral changes in rats
1	No body movement or only simple head movement in rats
2	The abdominal muscles of rats started to contract
3	The lower abdominal wall of the rat is lifted off the bottom of the box or flattened by significant contraction
4	The abdominal wall of the rat is arched or with the body and pelvis bowed

### Stool trait score

The faecal trait score was measured using the Bristol faecal typing score to evaluate faecal traits (Lewis and Heaton 1997). Briefly, the rat's faeces are scored as follows: 1: scattered hard lumps, nut-like; 2: salami-like, but in lumps; 3: salami-like, but with surface cracks; 4: salami-like or snake-like, smooth and soft; 5: soft mass with clear edges; 6: fluffy material with unclear edges, paste-like stool; 7: watery, no solid material.

### Fecal water content

The faeces produced by each rat were collected after being left in an empty container for 2 h, weighed in aluminium foil shortly after collection and then remeasured after drying at 80 °C overnight. The percentage of water was derived from the ratio of dry weight/prebaked weight. The water content of the faeces was calculated as follows: Water content of faeces = (wet weight of faeces—dry weight of faeces)/wet weight of faeces × 100%.

### Hematoxylin-eosin staining

Sequentially put the sections into xylene-I for 20 min—xylene II for 20 min—anhydrous ethanol I for 5 min—anhydrous ethanol II for 5 min—75% alcohol for 5 min, rinse with water. The sections were stained with hematoxylin staining solution for 3–5 min, washed with distilled water, divided with fractionation solution, washed with distilled water, returned to blue with blue return solution and rinsed with running water. The slices were then dehydrated in 85% and 95% gradient alcohol for 5 min each, and stained in eosin staining solution for 5 min. They were placed in anhydrous ethanol I for 5 min—anhydrous ethanol II for 5 min—anhydrous ethanol III for 5 min—dimethyl I for 5 min—xylene II for 5 min for transparency, and sealed with neutral gum. Microscopic examination (Nikon, Nikon Eclipse E100, Japan), image acquisition and analysis.

### Immunofluorescence staining

The sections were placed in xylene-I for 15 min—xylene-II for 15 min—anhydrous ethanol I for 5 min—anhydrous ethanol II for 5 min—85% alcohol for 5 min—75% alcohol for 5 min—distilled water wash in sequence. Tissue sections were placed in a repair cassette filled with citric acid antigen repair buffer (pH 6.0) for antigen repair in a microwave oven. Boil over medium heat for 5 min. The blocking solution is gently shaken off and a proportion of primary antibodies ZO-1 (BOSTER PB9234, Wuhan, China) and MUC2 (BOSTER BM5029, Wuhan, China) in PBS is added dropwise to the sections, which are incubated overnight at 4 °C in a wet box. The slides were washed three times for 5 min each on a decolorised shaker in PBS (pH 7.4), shaken and dried, and then covered with a secondary antibody of the appropriate species in a circle and incubated for 50 min at room temperature, protected from light. Sections were observed under a Nikon positive fluorescence microscope and images were taken.

### Enzyme-linked immunosorbent assay (ELISA)

Using proteins extracted from plasma and colon tissue, IL-1β, IL-6, IL-10, TNF-α, (BOSTER, Wuhan, China), IL-8, IgA (NEOBIOSCIENCE, Shenzhen, China) levels were measured according to the kit instructions.

### Cytokine gene microarrays

Gene expression profiles were analysed using the Rat Cytokine and Chemokine qPCR Array (Wcgene Biotech, Shanghai, China) according to the manufacturer's protocol, and data were analysed using Wcgene Biotech software. Genes with a fold change greater than or less than 2.0 were considered biologically significant.

### Real-time quantitative PCR

Detection of relative expression of Muc2, ZO-1, occluding (OCLN), claudin4 (CLDN4) related genes in colon tissue. Total RNA was extracted from colon tissue using Trizol (TIANGEN, Beijing, China) and cDNA was synthesized using FastQuant cDNA first-strand Synthesis Kit (TIANGEN, Beijing, China). quantitative RT-PCR was performed on ABI QuantStudio 6 using SYBR Green master mix (TIANGEN). The primer sequences used are shown in Table 2 (Primers from GENERAL BIOL). All samples were normalized to β -actin and the fold change in expression was calculated using the ΔΔCΤ method.

**Table 2:** The primers used in this experiment

Gene Name	Primer Sequence (from 5' End to 3' End)	Product Size (bp)
β-actin-F	CACCATGTACCCAGGCATTG	173
β-actin-R	CCTGCTTGCTGATCCACATC	
Muc2-F	TCCACCTACGGAGTCCACTA	161
Muc2-R	CTGAAGATGTGGTGGGTCCT	
ZO-1-F	AAATGACCGAGTCGCAATGG	199
ZO-1-R	GTGCACATCCTCGTCATAGC	
OCLN-F	CTACGGAGGGTACACAGACC	178
OCLN -R	CACCATGATGCCCAGGATTG	
CLDN4-F	CTGTGGATGTCCTGCGTTTC	125
CLDN4-R	CCCAGCAGGATGCCAATTAC	

### Microbiota analysis by 16S sequencing

Total genome DNA from cecal contents were extracted using Magnetic Soil And Stool DNA Kit (TIANGEN). DNA concentration and purity were monitored on 1% agarosegels. According to the concentration, DNA was diluted to 1 ng/μl using sterile water. PCR amplification of selected V3-V4 variable regions was performed using specific primers with Barcode and high fidelity DNA polymerase according to the selection of sequencing regions. PCR products were examined by 2% agarose gel electrophoresis and the target fragments were recovered by gel cutting using the AxyPrepDNA Gel Recovery Kit (AXYGEN). The PCR amplified recovered products were detected and quantified by reference to the preliminary quantification results of electrophoresis using the QuantiFluor™ -ST Blue Fluorescence Quantification System (Promega), and mixed in the appropriate proportions according to the sequencing volume required for each sample. Libraries were constructed using the NEB Next® Ultra™ DNA Library Prep Kit. Libraries were quality checked by Agilent Bioanalyzer 2100 and Qubit and sequenced after passing the library quality check (Detailed experimental and statistical methods are shown in Additional file 1).

### Short-chain fatty acids

SCFA detection was performed by gas chromatography-mass spectrometry (GC–MS). A mixture of acetic acid, propionic acid, butyric acid, isobutyric acid, valeric acid, isovaleric acid, hexanoic acid, isohexanoic acid and 2-ethylbutyric acid was prepared, and then prepared into 0.1 μg/mL, 0.5 μg/mL, 1 μg/mL, 5 μg/mL, 10 μg/mL, 50 μg/mL, 100 μg/mL. The samples were pretreated and subjected to GC–MS (Agilent Technologies Inc. CA, UAS), and the peak areas of the substances in the samples to be tested were calculated based on the plotted standard curves and the relative peak areas of the samples. The content of the substance in the sample was calculated based on the standard curve and the peak area of the substance in the sample. The default parameters of Masshunter quantification software (Agilent, v10.0.707.0, USA) were used to automatically identify and integrate the ionic fragments of the target short-chain fatty acids, with the aid of manual checks. The actual content of short-chain fatty acids in the sample was converted by calculating the detection concentration of each sample from the standard curve (Detailed experimental and statistical methods are shown in Additional file 1).

### Association between microbiota and cytokines and tight junctions (TJs)

The TOP10 flora at the genus level in the RS group of intestinal flora were correlated with inflammatory factors and tight junctions, and Spearman was chosen for the correlation calculation.

### Data analysis

Results are expressed as mean ± standard deviation, and comparisons between groups were made using one-way analysis of variance (ANOVA) and Tukey's multiple comparison test, and unpaired t-tests were used to compare differences between the two groups (GraphPad Prism version 8.0), with a p-value of 0.05 being considered significant.

## Results

### Basic indicators

#### General condition

After modelling, the rats in CS group and RS group showed clustering, loss of appetite, coarse hair, weight loss and perianal filth, while in the CS group the faeces were well formed but thin and soft, and in the RS group a clearly unformed and thin faeces could be seen (Fig. 1B-F). Body weight increased in all groups after 14 days of moulding and decreased in all groups after 28 days, with statistically significant differences (Fig. 1G).

#### Visceral hypersensitivity

**Fig. 2:**
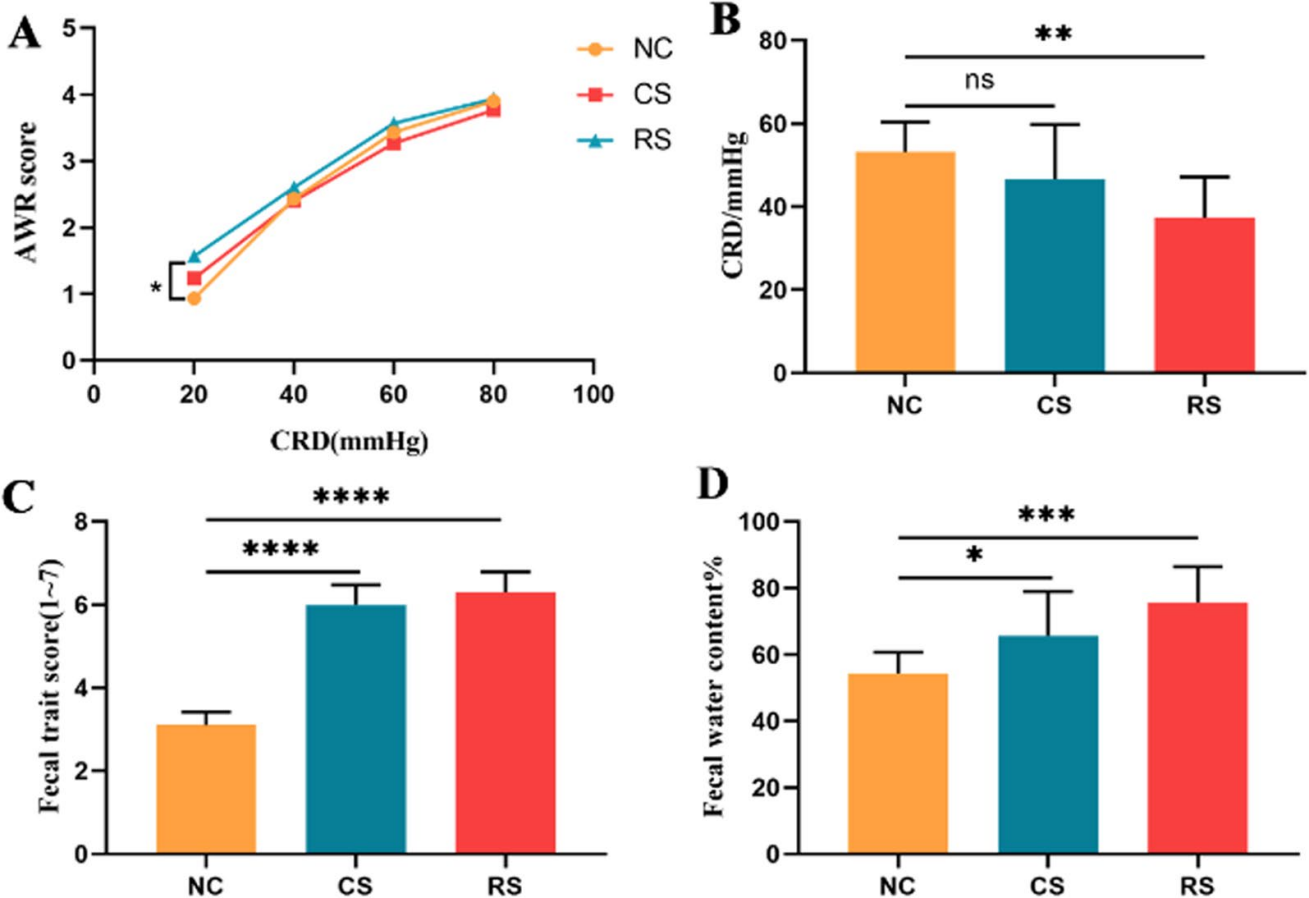
Behavioural evaluation of rats. **A** Rat AWR score; **B** Comparison of pain thresholds; **C** Fecal trait score; **D** Fecal water content. *p < 0.05, ** p < 0.01, *** p < 0.001, **** p < 0.0001

The RS group scored significantly higher than the NC group for the same CRD pressure in the AWR range of 20–40 mmHg, and the CS group scored higher than the NC group but the difference was not statistically significant. There was no difference between the NC, CS and RS groups in the CRD at 80 mmHg; there was a difference between the RS groups in each pressure range of the CRD (p < 0.05), showing a trend towards higher scores at higher pressures, but no significant difference between the 80 mmHg and 60 mmHg (p> 0.05) (Fig. 2A). Pain thresholds were lower in both the CS and RS groups compared to the NC group, but the difference was statistically significant in the RS group and not in the CS group (Fig. 2B).

#### Changes in stool characteristics

Compared with the NC group, the fecal trait scores and fecal water content were higher in the RS and CS groups (p < 0.05). The fecal trait score and fecal water content were higher in the RS group than in the CS group, but the difference was not statistically significant (p > 0.05) (Fig. 2C, D).

### Intestinal mucosal barrier dysfunction

**Fig. 3:**
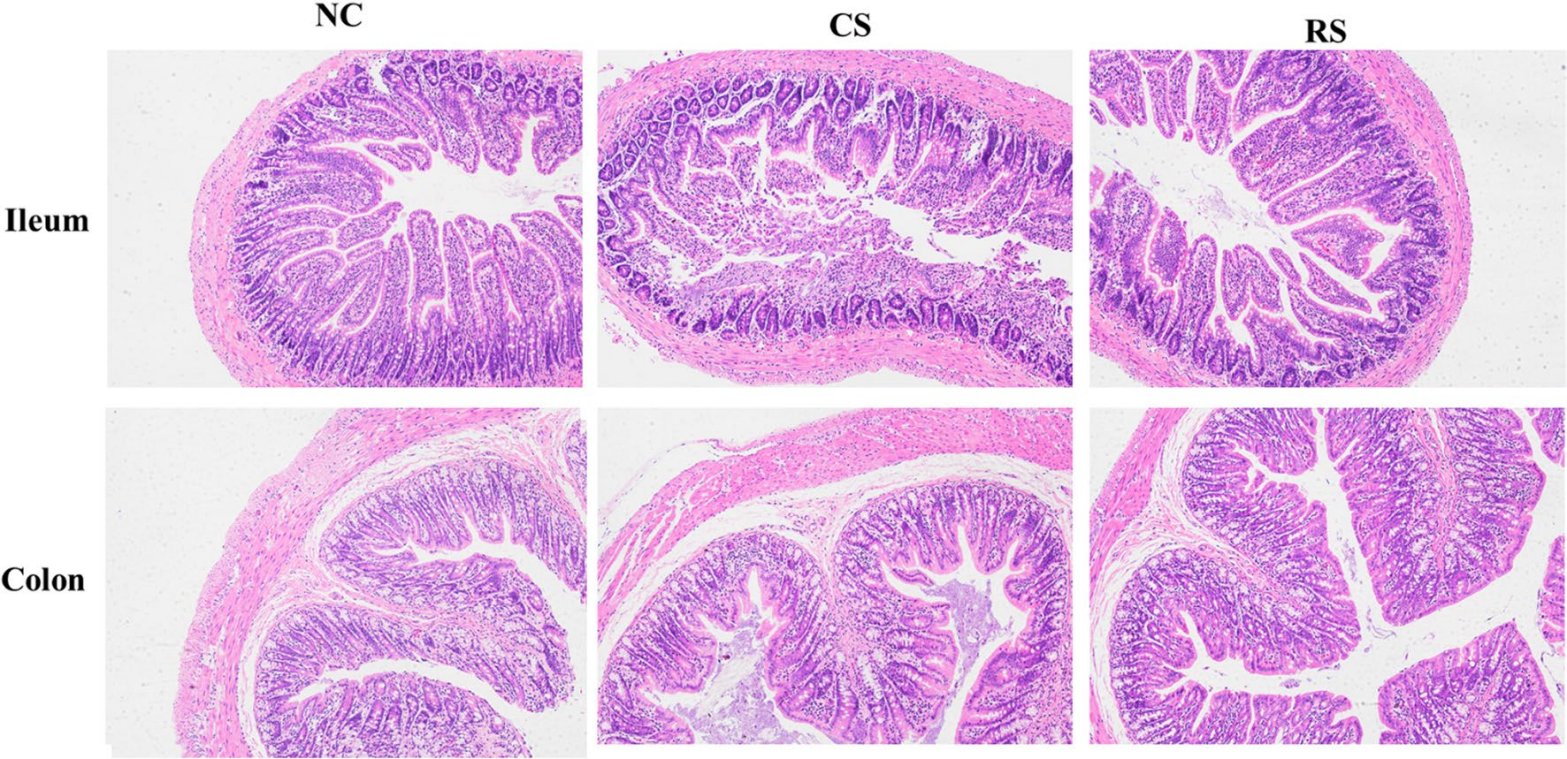
Representative hematoxylin–eosin staining of ileal and colonic tissues of various groups of rats (200X)

In the NC and RS groups, the ileum and colon were structurally intact and morphologically normal, with neatly arranged glands, tightly packed crypt foci and evenly distributed cup cells, and no damage was observed. In the CS group, the intestinal tissue integrity was destroyed, the boundary of each layer was not obvious, the intestinal mucosa was necrotic and detached, and the intestinal wall was significantly thickened, accompanied by different degrees of congestion, edema and inflammatory cell infiltration (Fig. 3).

**Fig. 4:**
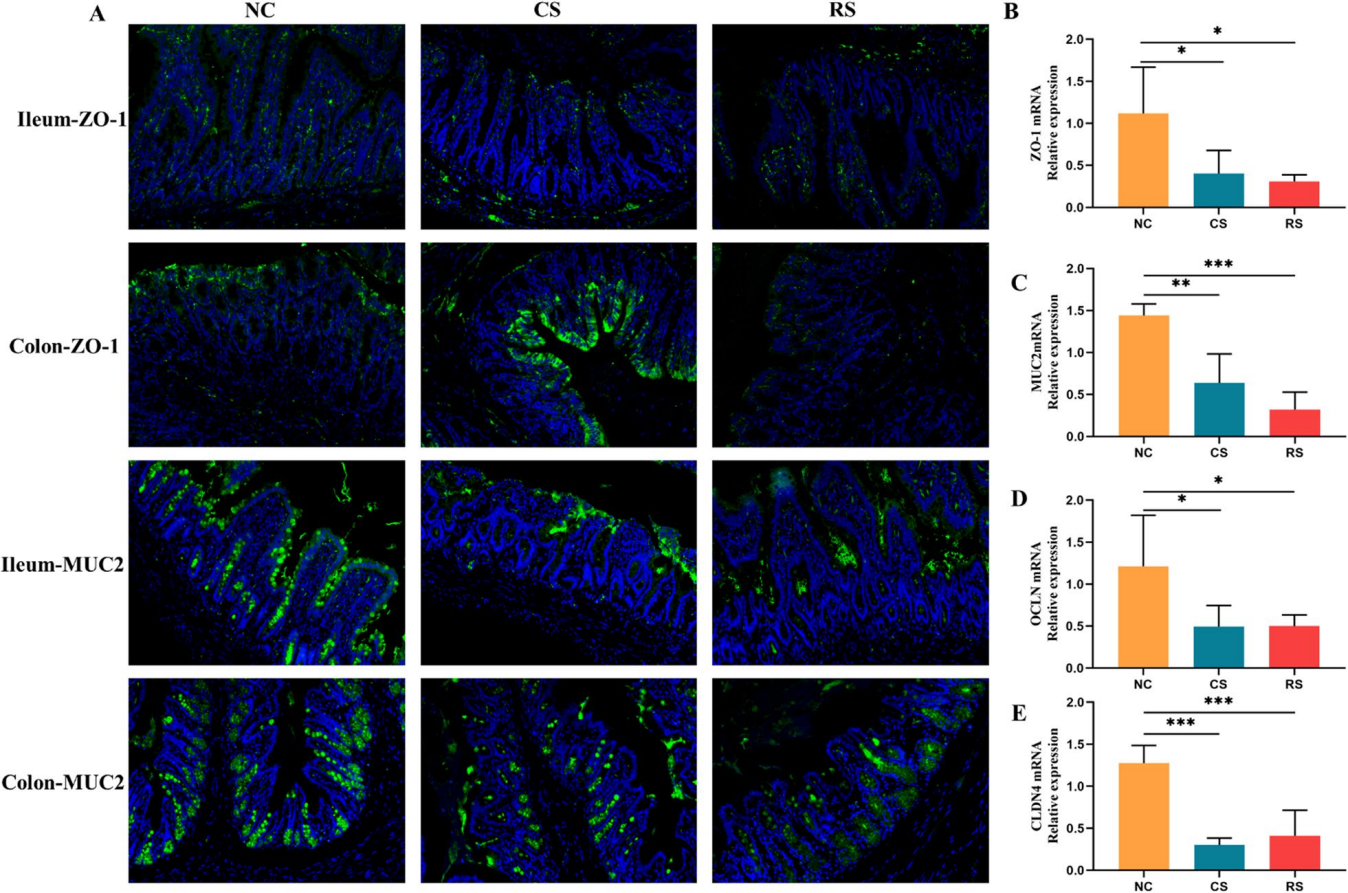
Intensity of ZO-1 and MUC2 (green) in ileal and colonic tissues of rats in each group. Nuclei stained with DAPI (blue) (200×) (**A**), mRNA expression levels of ZO-1 (**B**), MUC2 (**C**), OCLN (**D**), CLDN4 (**E**) in each group of rats.* p < 0.05, ** p < 0.01, *** p < 0.001

Immunofluorescence results showed that ZO-1 and MUC2 were mainly expressed in the epithelial layers of the ileum and colonic mucosa. The expression of ZO-1 and MUC2 was significantly reduced in the CS and RS groups compared to the NC group. RT-PCR results showed that the expression levels of MUC2, ZO-1, OCLN and CLDN4 mRNA were reduced in the CS and RS groups, and the differences were statistically significant (Fig. 4).

### Weakened immune regulation

**Fig. 5:**
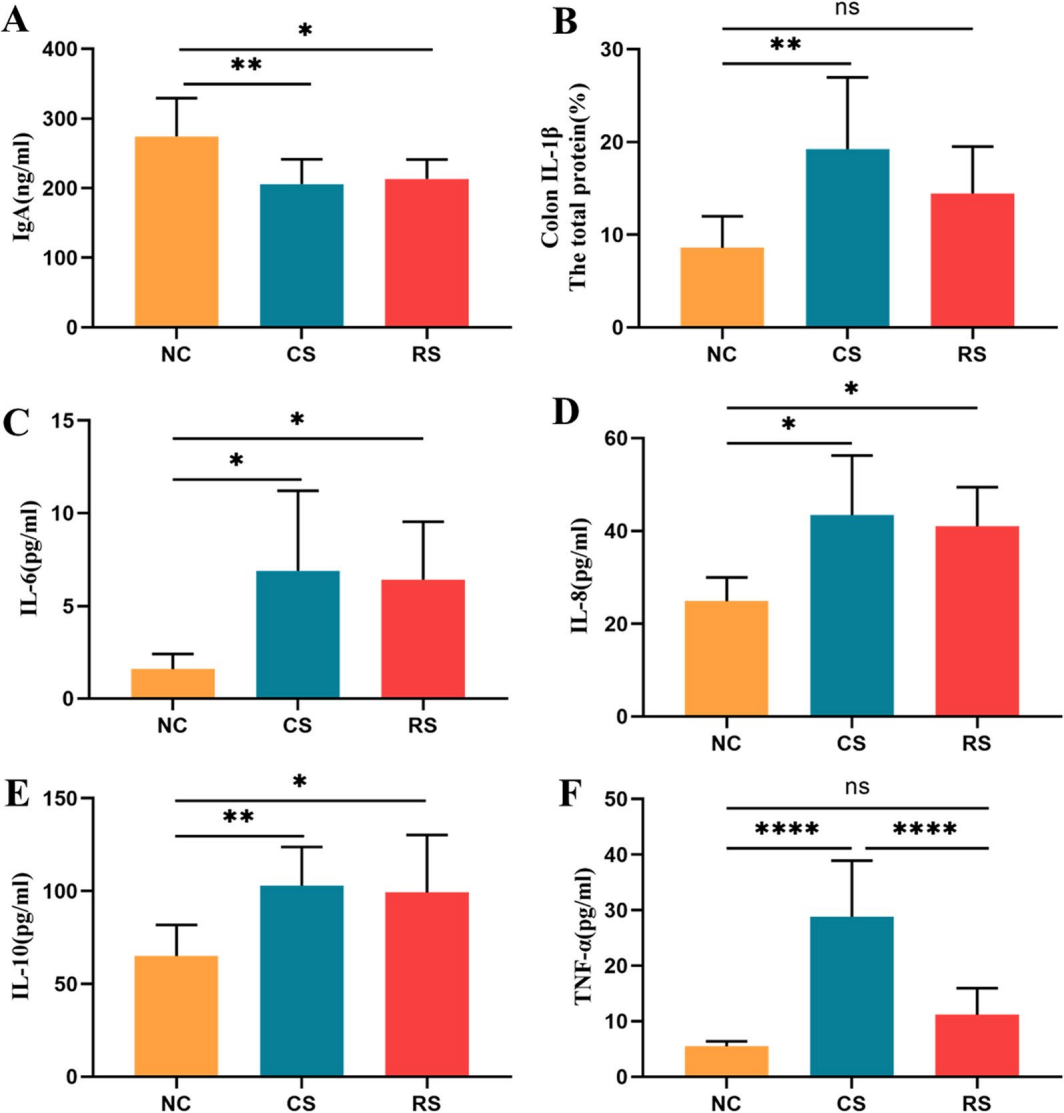
Effects of IgA, IL-1β, IL-6, IL-8, IL-10 and TNF-α in plasma and colonic tissues of rats. The expression levels of IgA (**A**), IL-6 (**C**), IL-8 (**D**), IL-10 (**E**) and TNF-α (**F**) in plasma and IL-1β (**B**) in colon tissues were determined using ELISA. * p < 0.05, ** p < 0.01, *** p < 0.001, **** p < 0.0001

IgA, IL-1β, IL-6, IL-8, IL-10, TNF-α levels were measured using ELISA. IgA expression was significantly lower in the CS and RS groups than in the NC group, and IL-1β, IL-6, IL-8, IL-10, TNF-α expression was significantly greater in the CS and RS groups than in the NC group (p < 0.05) (Fig. 5A–F).

**Fig. 6:**
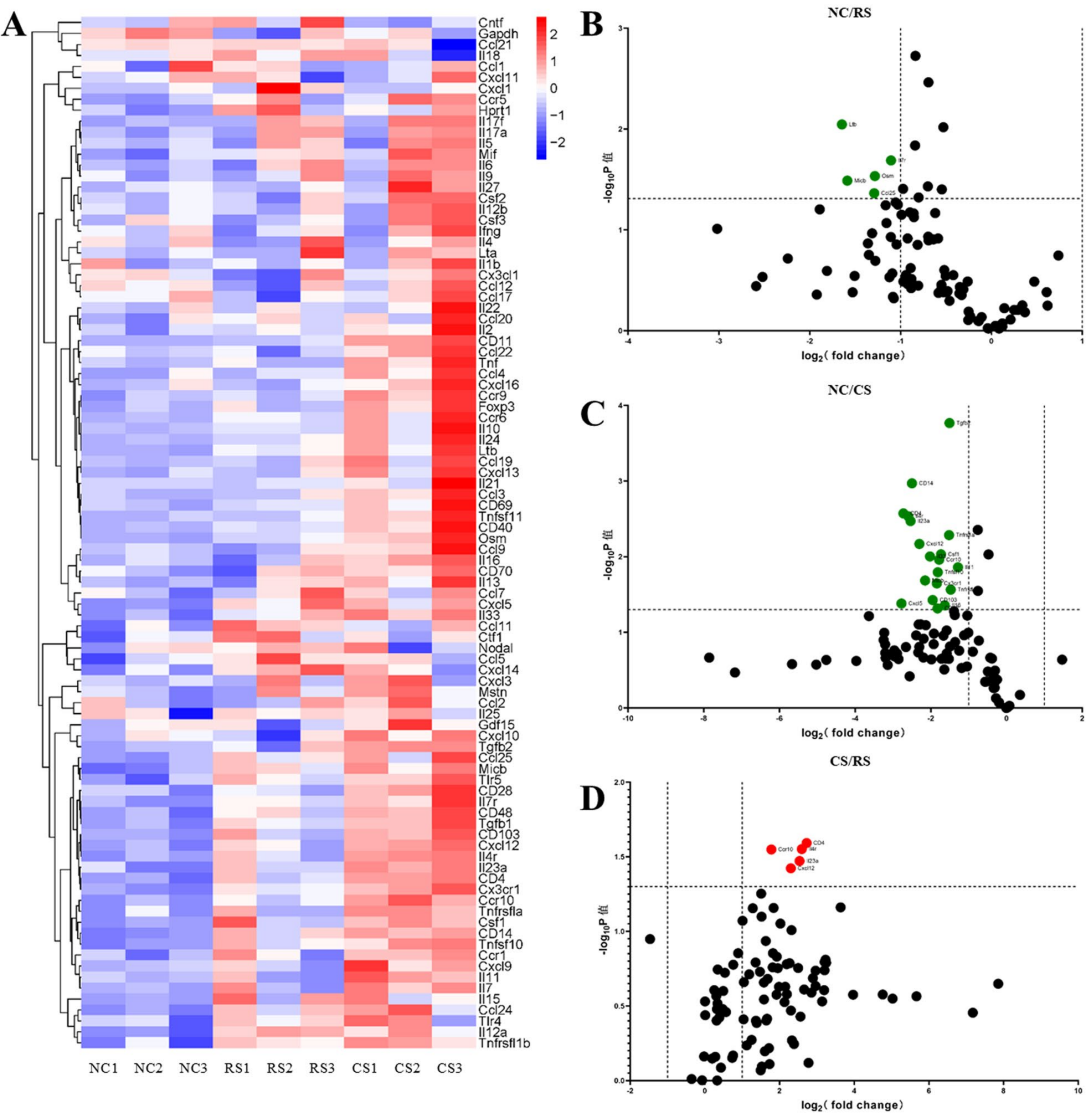
Cytokine profiles in rat colon tissue. **A** Hierarchical clustering represents cytokine mRNAs that differ significantly between groups (fold-change > 2, p < 0.05), and in the heat map, red indicates high expression and blue indicates low expression. **B**, **D** Volcano plot showing differential expression of multiple mRNAs in colonic tissue between groups

Cytokine and chemokine RT-PCR was applied to analyse changes in the expression profiles of cytokines in the colonic mucosa of the RS and CS groups. The heat map represents the hierarchical clustering of the three groups of differentially expressed cytokine genes (Fig. 5A), in which high expression is shown in red and low expression is shown in blue. Volcano mapping showed that the genes that differed between the RS and CS groups were: chemokine (CCL) 25, leukotriene B (LtB), interleukin (IL) 17r, MHC class I chain-related gene product (MICB), and oncoprotein M (OSM) (Fig. 6B). The genes that differed between the CS and NC groups were Ccr10, Cxcl12, Cxcl5, clusters of differentiation(CD) 103, CD14, CD4, CD48, colony-stimulating factor 1(CSF1), IL16, IL23α, IL4r, MICB, transforming growth factor-β2, tumor necrosis factor ligand superfamily (Tnfsf)10 (Fig. 6C). mRNA expression of CD4, IL-4R, IL-23α, CCR10, CXCL12 differed between the RS and CS groups (Fig. 6D).

### The microbial signature of different diarrhea-predominant irritable bowel syndrome models

For sequencing analysis of the intestinal flora of cecum contents, the dilution curve and species accumulation curve reflect the adequacy of sequencing data volume and indirectly reflect the abundance of species in the sample. The flatness of the curve evaluates whether the sequencing sample volume is sufficient, and when the curve tends to be flat, it indicates that the sequencing data volume is sufficient. As shown in Fig. 8A and B, the dilution curve is flat, indicating that the amount of data obtained from this sequencing meets the sequencing requirements and can represent the majority of microbial information contained in the sequenced samples.

**Fig. 7:**
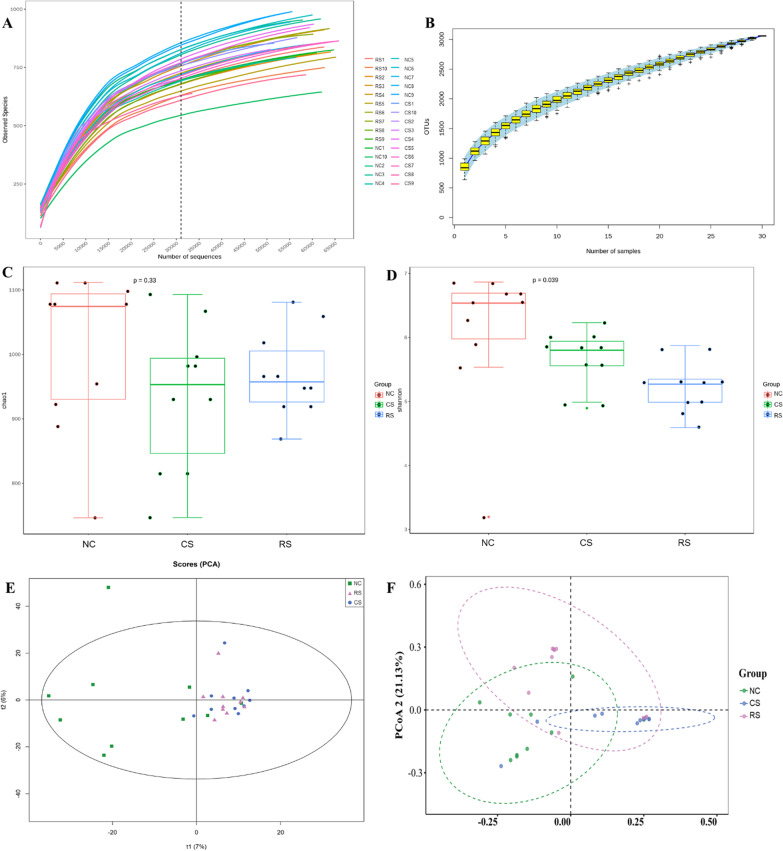
Differences in intestinal metabolites between groups of rats. **A** Rarefaction Curve, **B** Species Accumulation Curves, α Diversity Index Intergroup Difference Analysis: **C** Chao, **D** Observed Species. β Diversity: **E** Principal Component Analysis, **F** PCoA, Principal Co-ordinates Analysis

OTU-based alpha diversity analysis refers to the diversity in a specific region or ecosystem, and commonly used measures are Chao1, shannon etc. Compared to the NC group, Chao1 decreased slightly in the RS and CS groups, but the difference between the two groups was not statistically significant (Fig. 7C). This indicates that the differences in community richness among the three groups were not significant. Compared to the NC group, shannon was significantly lower (P < 0.05) in the RS and CS groups (Fig. 7C). This indicates a significant difference in community diversity between the three groups. Principal component analysis and principal coordinate analysis showed that there was some variability between the NC, CS and RS groups (Fig. 7E, F).

**Fig. 8:**
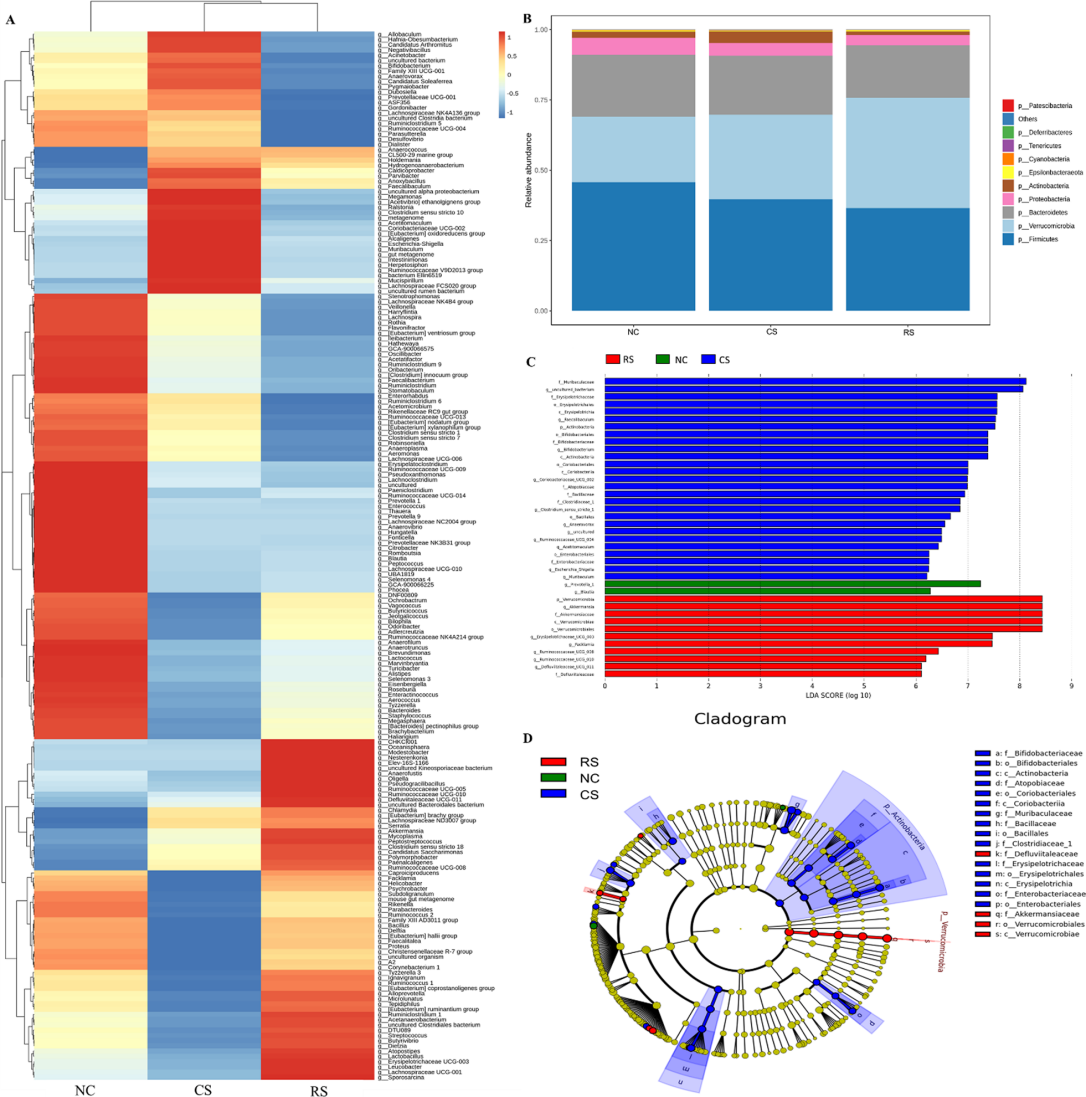
Gut flora composition of IBS-D rats. **A** Heat map analysis of species richness clustering at the genus level, **B** histogram of species richness at the phylum level for each group of samples, **C** Linear discriminant analysis(LDA) Score, **D** evolutionary branching diagram

**Fig. 9:**
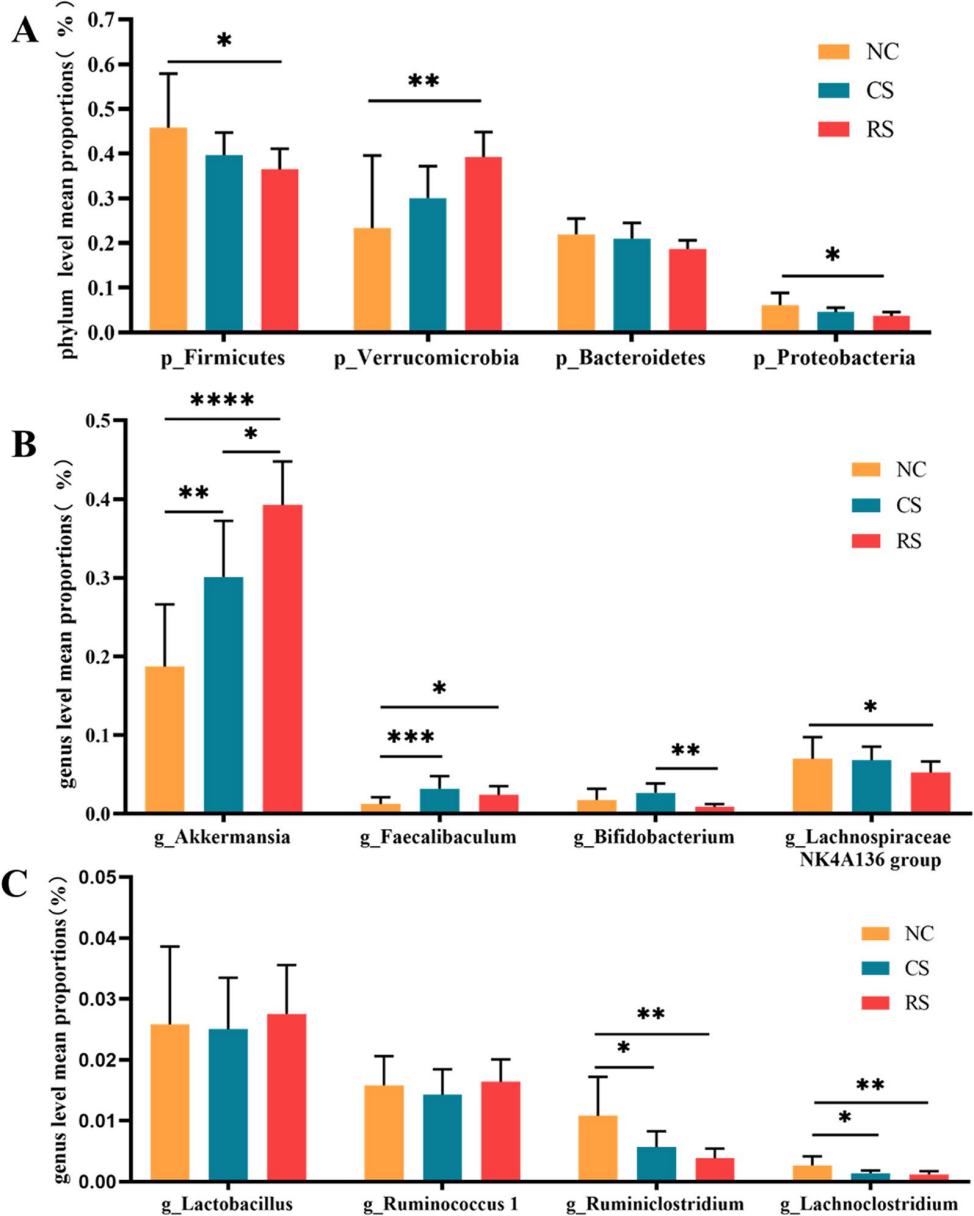
Top bacterial rankings of microbial abundance at the phylum and genus level. **A** Phylum level, **B**, **C** genus level. * p < 0.05, ** p < 0.01, *** p < 0.001, **** p < 0.0001

At the phylum level, Compared with the NC group, the RS group had lower relative abundance of *Firmicutes* (P < 0.05), higher relative abundance of *Verrucomicrobia* (P < 0.05), and reduced relative abundance of *Proteobacteria* and *Bacteroidetes*, but there was no statistical difference. The CS group had a lower relative abundance of *Firmicutes, Proteobacteria,* and *Bacteroidetes* decreased and *Verrucomicrobia* increased, all differences were not statistically significant (Figs. 8B, 9A).

At the genus level, *Bifidobacterium, Ruminiclostridium, Rumenococcus, lachnospiraceae_nk4a136_group* and *Lachnoclostridium* were reduced and *Akkermansia* and *Faecalibaculum* were increased in the RS group compared to the NC group. Compared with the CS group, the RS group showed an increase in *Akkermansia* and a decrease in *Bifidobacterium* and *Faecalibaculum* at the genus level (Figs. 8A, 9B, C).

The abundance scores of different levels of Linear discriminant analysis (LDA) taxa proved that the RS groups *Verrucomicrobia, Akkermansiaceae, Erysipelotrichaceae, Erysipelotrichaceae, facklamia, ruminococcaceae_UCG_008, ruminococcaceae_UCG_010*, *defluviitaleaceae* species richness. CS group *muribaculaceae, uncultured Bacterium, faecalibacterium, Erysipelotrichaceae* Species richness was high (Fig. 8C).

The results of bacterial alterations in the taxonomic evolutionary dendrograms obtained from Linear discriminant analysis Effect Size (LEfSe) showed that the microbiota with important roles in the RS group were Verrucomicrobia, defluviitaleaceae, Akkermansiaceae, and the microbiota with important roles in the CS group were Bifidobacteriaceae, Erysipelotrichaceae, Enterobacteriaceae, Actinobacteria, Bacillaceae (Fig. 8D).

### SCFAs

**Fig. 10:**
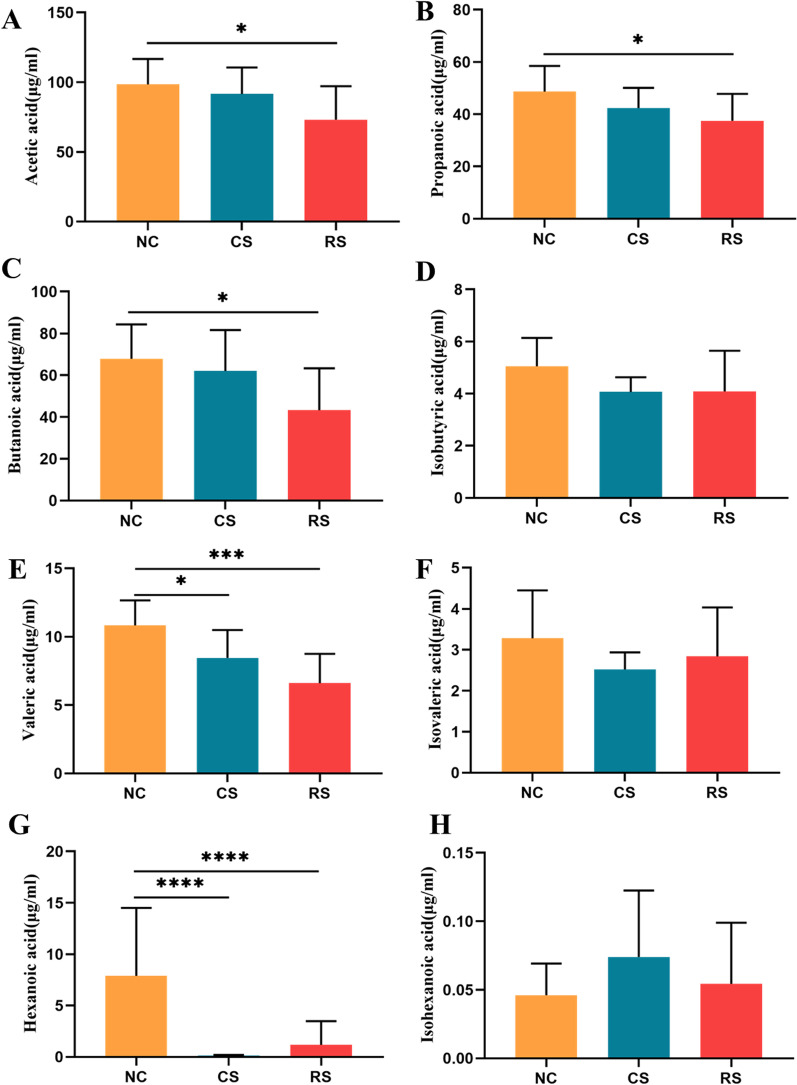
Changes in short-chain fatty acid concentrations in various groups of rats. **A** Acetic acid, **B** Propionic acid, **C** Butyric acid, **D** Isobutyric acid, **E** Valeric acid, **F** Isovaleric acid, **G** hexanoic acid, **H** Isohexanoic acid, * p < 0.05, ** p < 0.01, *** p < 0.001, **** p < 0.0001

The analysis of short-chain fatty acids in the cecum contents revealed that; compared with the NC group, the concentrations of acetic acid, propionic acid, butyric acid, valeric acid and hexanoic acid in the cecum contents of RS rats were significantly reduced (P < 0.05), while the concentrations of isobutyric acid, isovaleric acid and isohexanoic acid were reduced but the difference was not significant (P > 0.05). The concentrations of valeric acid and hexanoic acid were significantly reduced in the CS group, and the concentrations of acetic acid, propionic acid, butanoic acid, isobutyric acid, isovaleric acid, and isohexanoic acid were slightly reduced compared with the NC group, with no statistical difference (P > 0.05) (Fig. 10A–H).

### Association between microbiota and cytokines and tight junctions (TJs)

**Fig. 11:**
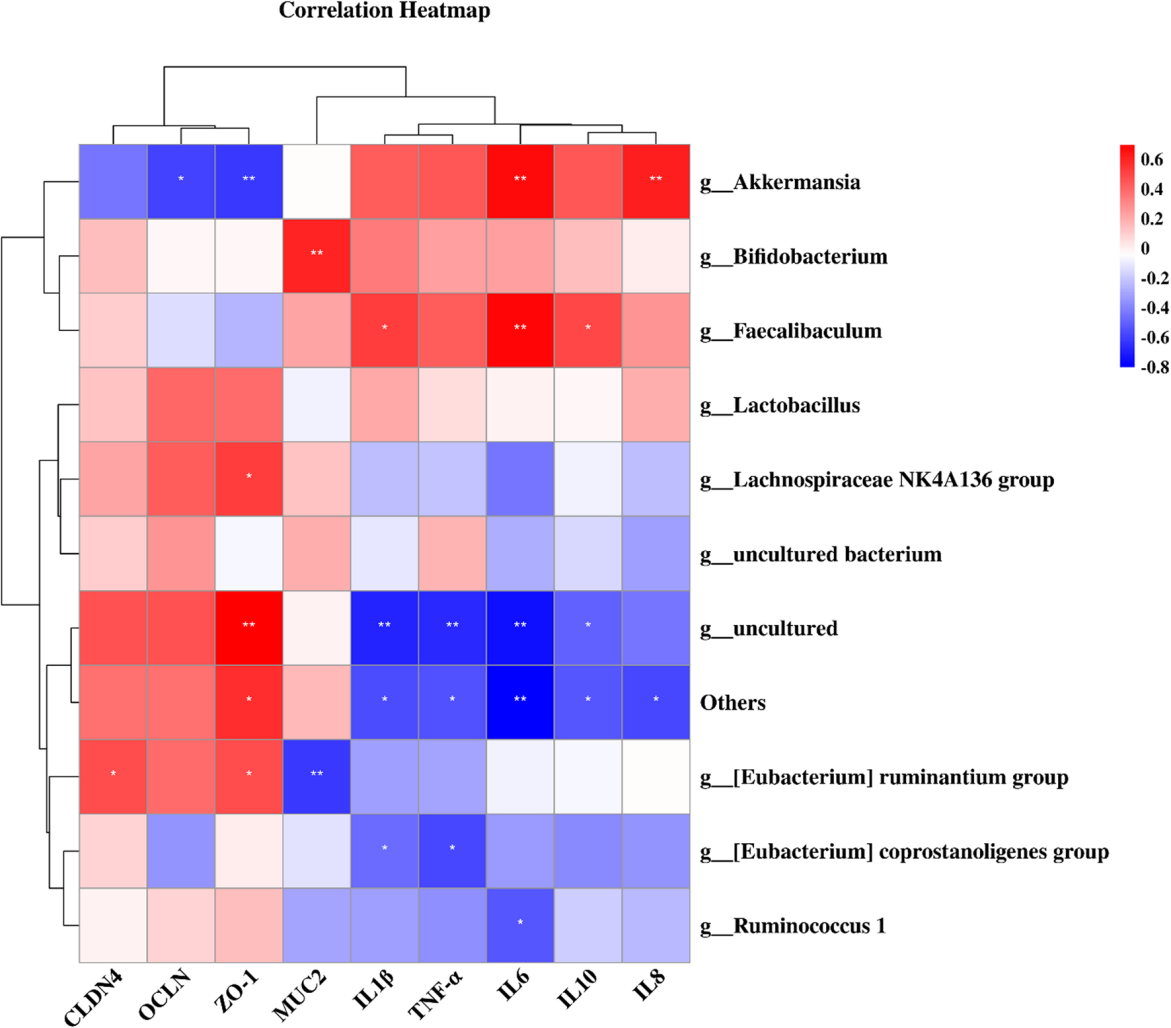
Correlation analysis heatmap focusing on the association between microbiota and TJ, cytokines. Scale (right legend) indicates the level of positive (red) or negative (blue) correlation, and asterisks indicate significance. *p < 0.05, **p < 0.01, ***p < 0.0001

To further elucidate the underlying mechanisms, we assessed the association between representative values of TJs, cytokines, and intestinal flora changes in rats (Fig. 11). In the RS group, *Akkermansia* was positively correlated with inflammatory factor expression and negatively correlated with ZO-1, OCLN, and CLDN4 expression. Increased *Akkermansia* abundance was associated with higher IL6, IL8 expression and lower ZO-1, OCLN, and CLDN4 expression. Decreased *Bifidobacterium* abundance was associated with muc2 expression was correlated with increased MUC2 expression.

*Faecalibaculum* was positively correlated with inflammatory factors, and an increase in *Faecalibaculum* abundance was associated with higher expression of IL1, IL6 and IL10.In addition, *Ruminantium group*, *Coprostanoligenes group* and *Ruminococcus* were negatively correlated with inflammatory factors and positively correlated with TJs. In conclusion, these results provide possible evidence for a link between intestinal ecological dysregulation, immune dysregulation, intestinal barrier dysregulation, and diarrhea in IBS-D.

## Discussion

The results of this study indicate that both chemical stimulation and restraint stress can increase the excessive visceral motor response to CRD in rats with significant diarrheal symptoms and weight loss. The decrease in mucin secretion and tight junction content, weakened intestinal mucosal barrier function, increased inflammatory factor content, decreased content of beneficial bacteria such as *Bifidobacteria,* and different degrees of reduction in SCFAs suggest that the occurrence of IBS-D is closely related to visceral hypersensitivity, immune regulation, intestinal flora, and metabolites. Visceral hypersensitivity and fecal water content after stress stimulation are higher than chemical stimulation, suggesting that psychological or physical stress is an important risk factor for the development and progression of IBS (Mayer et al. 2001; Walker et al. 2008; Mujagic et al. 2022). High stress affects the microbiota leading to imbalance in the gut-brain axis, a weakened immune system, impairment of the intestinal barrier, limitation of water absorption in the gastrointestinal tract, and ultimately diarrhea (Scuderi et al. 2020; Blake et al. 2016; Schaper and Stengel 2022; Galley et al. 2014; Moser et al. 1946; Ancona et al. 2021). In comparison, the changes in visceral hypersensitivity, intestinal barrier function and intestinal flora in the stress stimulation combined with chemical stimulation model were closer to the pathological characteristics of IBS-D patients, suggesting that stress may be a key factor affecting it.

Stress may lead to disruption of TJs, damage to the intestinal mucosal barrier, and decreased intestinal resistance to pathogens, ultimately triggering IBS-D (Zhang et al. 2021a; Camilleri et al. 2012; Hou et al. 2019; Sánchez de Medina et al. 2014). It has been shown that intestinal permeability is increased and ZO-1 and ocludin expression is reduced in patients with IBS-D and animal models (Sánchez de Medina et al. 2014; Dunlop et al. 2006). In this study, intestinal mucosal barrier function was assessed by measuring MUC2, ZO-1, and ocludin, and intestinal TJs mRNA expression was significantly lower in the RS group compared with the NC group, and this downregulation may be closely related to microRNA-144 (miR-144), which is significantly upregulated and leads to downregulation of ocludin and ZO-1 expression (Hou et al. 2017).

Stress can stimulate the production of inflammatory cytokines by T lymphocytes through mast cell products, disrupting the mucosal barrier and increasing intestinal permeability (Demaude et al. 2006). Our study found that the expression of inflammatory factors (IL-1β, IL-6, IL-8, IL-10, TNF-α) was elevated in the RS group. Some studies have shown elevated pro-inflammatory cytokines (IL-6, TNF-α) and decreased anti-inflammatory cytokine IL-10 in patients with IBS (Mitselou et al. 2020; Kumar et al. 2022), while others have shown no difference between patients with IBS and healthy controls (Kamp et al. 2021). Although changes in inflammatory cytokine levels in IBS have been inconsistently reported, it has been shown that changes in cytokines may be associated with changes in the gut microbiota (Hustoft et al. 2017; Zhou et al. 2022).

The gut microbiota can influence intestinal and central nervous system (CNS) disorders, and dysbiosis of the gut flora has been shown to be an important mechanism in the pathogenesis of IBS (Shamsipour et al. 2021; Hillestad et al. 2022).Our study showed a decrease in microbial diversity in the intestinal contents of IBS-D rats, with a decrease in *Bifidobacterium, Lachnospira, Ruminococcus, lachnospiraceae,* and an increase in *Akkermansia* and *Faecalibaculum*,in agreement with the findings already reported (Zhou et al. 2022; Mazzawi et al. 2019; Fu et al. 2022). The overall trend of bacterial flora changes was similar between the CS and RS groups, but the relative abundance of *Verrucomicrobia* and *Akkermansia* species was higher and the relative abundance of *Firmicutes, Bacteroidetes, Bifidobacterium* and *Faecalibaculum* species was lower in the RS group, which indirectly indicates that stress stimulation is more capable of causing changes in the gut microbiota.

Haomeng Wu et al. observed a close relationship between gut microbiota and brain-gut peptides (5-HT, CRF, NPY), and that the gut microbiota appears to be a key mediator of messages in brain-gut dialogue, with bidirectional signals sent between the CNS and the gastrointestinal tract influencing the stress response (Strandwitz 2018; Wu et al. 2022). *Bifidobacterium* have a positive effect on the central nervous system, reducing stress-related visceral hypersensitivity through synergistic hypothalamic–pituitary–adrenal axis regulation, and a decrease in *Bifidobacterium* may lead to increased intestinal sensitivity (Ait-Belgnaoui et al. 2018). When the intestine is stimulated, 5-HT increases, making the enteric nervous system and visceral afferent nerves highly sensitive, leading to discomfort, abdominal pain, and diarrhea (Chen et al. 2022).

We clustered representative values of intestinal flora changes with tight junctions and cytokines and found that *Akkermansia* was negatively correlated with the expression of tight junctions and positively correlated with the expression of inflammatory cytokines. *Akkermansia muciniphila* was shown to be a human intestinal mucin-degrading bacterium (Derrien et al. 2004) that can use host-secreted mucus glycoproteins as source of nutrition, leading to erosion of the colonic mucus barrier. Although the intestinal barrier-protective and inflammation-reducing effects of *Akkermansia* have been reported in many studies, the results of Wang K et al. showed an increase in *Akkermansia* in pathological states, which is consistent with our results (Wang et al. 2022; Huang et al. 2022). Meanwhile, the controversy over the anti-inflammatory or anti-inflammatory role of *Akkermansia* was reviewed in a study (Zhang et al. 2021b). Excessive amounts of *Akkermansia* survive by excessive depletion of mucin. In this condition, the number of non-mucus-consuming species is significantly reduced, leading to reduced species diversity and abnormal *Akkermansia* proliferation, which may lead to disruption of the mucosal layer, reduced TJs, and damage to the intestinal barrier, inducing intestinal inflammation (Desai et al. 2016).

Short-chain fatty acids (SCFAs) are the main metabolites of the intestinal flora, and imbalances in the intestinal flora of IBS patients lead to changes in SCFA (Morrison and Preston 2016; Xiao et al. 2021). Lactobacillus can produce lactic acid and acetic acid, and Veillonella can convert lactic acid to acetic acid and propionic acid, and IBS patients with high acetic acid or propionic acid levels exhibit more severe gastrointestinal symptoms (Tana et al. 2010). Butyric acid induces mucin synthesis, strengthens connections between epithelial cells, and downregulates gene expression in the gut-associated immune system, thereby preventing inflammation and intestinal mucosal damage, suggesting that reduced levels of SCFA production may lead to increased inflammation (Magnusson et al. 2020).

**Fig. 12:**
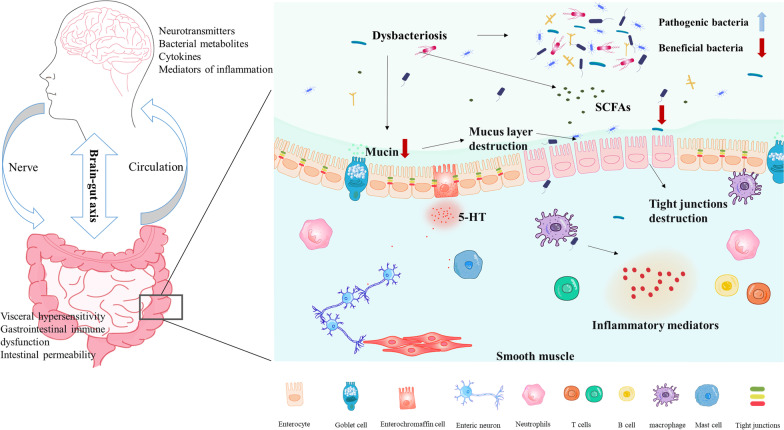
Pathogenesis of stress-induced irritable bowel syndrome

The pathogenesis of IBS-D involves changes in visceral hypersensitivity and intestinal permeability, which can be altered by intestinal microbes. The gut microbiota has a causal role in intestinal and behavioral manifestations, and imbalances in the gut flora of IBS patients affect the normal signaling interactions between short-chain fatty acids and intestinal epithelial cells, ultimately leading to an inflammatory response and increased intestinal epithelial barrier permeability triggering diarrhea (Mars et al. 2020; Crouzet et al. 2013; Palma et al. 2017) (Fig. 12).

A noteworthy point is that we found less harmful bacteria such as *Staphylococcus* in the NC group than in the RS group. This is inconsistent with the results of previous studies (Li et al. 2020) and may be due to the good anti-inflammatory and bactericidal effect of the chemical stimulus we used (rhubarb), which has a significant inhibitory effect on Staphylococcus, including its component rhubarbin (Yan et al. 2017). Régnier et al. found that rhubarb extract acted on the composition of the entire intestinal microbiota, especially on *Akkermansia muciniphila* genus (Régnier et al. 2020). In China, many studies have used rhubarb (Luo et al. 2021; Yj et al. 2017) or senna (Li et al. 2018) as a modeling method of choice for animal models of diarrhea. It is undeniable that excessive amounts of rhubarb can cause irritation hazards to the gastrointestinal tract and significant diarrheal symptoms, but its medicinal value should still not be underestimated. Herbs that can cause diarrhea such as rhubarb and senna have good bactericidal effects and can effectively inhibit the growth of harmful intestinal bacteria, so it is debatable whether this stimulatory approach to the IBS-D model is representative of the pathological state of the intestinal flora in patients with clinical IBS-D.

## Conclusion

Overall, the data obtained provide new insights into the choice of methods for the production of animal models of IBS-D. Restraint stimulation combined with chemical stimulation can mimic the pathological state of diarrhea symptoms, visceral hypersensitivity, reduced intestinal mucosal barrier permeability, immune regulatory dysfunction and dysbiosis in IBS-D patients. However, herbs with antimicrobial effects (e.g., rhubarb, senna, etc.) are not suitable as the first choice for chemical stimulation because they may lead to a decrease of harmful bacteria and an increase of beneficial bacteria in some parts of the intestine, which cannot perfectly simulate the imbalance of intestinal flora in IBS-D patients, and fasciculation stress may be a key factor in IBS-D modeling.

## Supplementary Information

**Additional file 1.** Detailed processes of intestinal flora and short-chain fatty acids.

**Additional file 2.** Extended figures of intestinal flora.

## Data Availability

The datasets used and analysed during the current study are available from the corresponding author on reasonable request.

## Abbreviations

IBS-D: Diarrheal irritable bowel syndrome
RS: Restraint stress
CS: Chemical Stimulation
NC: Normal control
AWR: Abdominal wall retreat test score
CRD: Colorectal dilatation
ELISA: Enzyme-linked immunosorbent assay
SCFAs: Short-chain fatty acids
IL: Interleukin
CCL: Chemokine
Muc2: Mucin 2
ZO-1: Zonulae occluden-1
OCLN: Occluding
CLDN4: Claudin4
TJs: Tight junction
LDA: Linear discriminant analysis
