# Supplementary material for: Evaluation of two laboratory model methods for diarrheal irritable bowel syndrome
Source: Mol Med. 2023 Jan 12;29:5. doi: 10.1186/s10020-022-00599-x (PMC9837933; doi:10.1186/s10020-022-00599-x)
Supplement: Supplementary file 1 — Additional file 1. Detailed processes of intestinal flora and short-chain fatty acids. [file 10020_2022_599_MOESM1_ESM.docx]

**Supplementary Data 1**

**1.Microbiota analysis**

Instrument

| Instrument | Company |
| --- | --- |
| Qubit@ 2.0 Fluorometer | Thermo Scientific |
| Agilent Bioanalyzer 2100 system | Agilent |
| Bio-rad T100 thermal cycler | Bio-rad |
| NovaSeq6000 | Illumina |

eagent consumables

| Reagent consumables | Company |
| --- | --- |
| Magnetic Soil And Stool DNA Kit | TIANGEN |
| Phusion®High-Fidelity PCR Master Mix | New England Biolabs |
| Qiagen Gel Extraction Kit | Qiagen |
| TruSeq® DNA PCR-Free Sample Preparation Kit | Illumina |

Primers

| Amplified Region/Primers Name | Foward primer sequences | Reverse primer sequences |
| --- | --- | --- |
| V3-V4 region / 341F-806R | CCTAYGGGRBGCASCAG | GGACTACNNGGGTATCTAAT |
| V4 region / 515F-806R | GTGCCAGCMGCCGCGGTAA | GGACTACHVGGGTWTCTAAT |
| ITS1region / 1737F-2043R | GGAAGTAAAAGTCGTAACAAGG | GCTGCGTTCTTCATCGATGC |
| ITS1region / ITS1F-ITS2 | CTTGGTCATTTAGAGGAAGTAA | GCTGCGTTCTTCATCGATGC |

**Method**

## Extraction of genome DNA

Total genome DNA from samples were extracted using Magnetic Soil And Stool DNA Kit (TIANGEN). DNA concentration and purity were monitored on 1% agarosegels. According to the concentration, DNA was diluted to 1ng/μl using sterile water.

## Amplicon Generation

16S rRNA genes were amplified used the specific primer with the barcode. All PCR reactions were carried out in 30 μL reactions with 15 μL of Phusion®High-Fidelity PCR Master Mix (New England Biolabs); 0.2 μM of forward and reverse primers, and about 10 ng template DNA. Thermal cycling consisted of initial denaturation at 98 ℃ for 1 min, followed by 30 cycles of denaturation at 98℃ for 10 s, annealing at 50 ℃ for 30 s, and elongation at 72 ℃ for 30 s. Finally 72 ℃ for 5 min.

## PCR Products quantification and qualification

Mix same volume of 1X loading buffer (contained SYB green) with PCR products and operate electrophoresis on 2% agarose gel fordetection. Samples with bright main strip between 400-450 bp were chosen for further experiments.

## PCR Products Mixing and Purification

PCR products was mixed in equidensity ratios. Then, mixture PCR products was purified with Qiagen Gel Extraction Kit (Qiagen).

## Library preparation and sequencing

Sequencing libraries were generated using TruSeq® DNA PCR-Free Sample Preparation Kit (Illumina) following manufacturer’s recommendations and index codes were added. The library quality was assessed on the Qubit@ 2.0 Fluorometer (Thermo Scientific) and Agilent Bioanalyzer 2100 system. At last, the library was sequenced on an Illumina NovaSeq6000 platform and 250bp paired-end reads were generated.

# Data analysis

## Paired-end reads assemblies

Paired-end reads from the original DNA fragments were merged using FLASH, a very fast and accurate analysis tool, which was designed to merge paired-end reads when at least some of the reads overlap the read generated from the opposite end of the same DNA fragment. Paired-end reads was assigned to each sample according to the unique barcodes.

## OTU cluster, species annotation and α-diversity

Sequences analysis were performed by UPARSE software package using the UPARSE-OTU and UPARSE-OTUref algorithms. In-house Perl scripts were used to analyze alpha (within samples) and beta (among samples) diversity. Sequences with ≥97% similarity were assigned to the same OTUs. We pick a representative sequences for each OTU and use the RDP classifier to annotate taxonomic information for each representative sequence (Silva 132 for 16S, UNITE for ITS). In order to compute Alpha Diversity, we rarify the OTU table and calculate four metrics: species abundance were estimated by Chao1 and Observed Species indexes, and microbial diversity were estimated by Shannon and Simpson indexes. Rarefaction curves were generated based on these metrics.

## Community composition and β-diversity

Graphical representation of the relative abundance of microbial composition from phylum to species can be visualized using Krona chart. Venn, barplot and heatmap were used to showing microbial community composition among samples and groups. Beta diversity were visualized through PCA, PCoA and NMDS. To identify differences of microbial communities among different groups, ANOSIM and adonis were performed based on the Bray-Curtis dissimilarity distance matrices.

## Statistical analysis

To confirm differences in the abundances of individual taxonomy between the two groups, STAMP software was utilized. Linear discriminant analysis Effect Size (LEfSe) was used for the quantitative analysis of biomarkers within different groups. This method was designed to analyze data in which the number of species is much higher than the number of samples and to provide biological class explanations to establish statistical significance, biological consistency, and effect-size estimation of predicted biomarkers.

**2.Short-chain fatty acids**

**2.1 Preparation of short-chain fatty acid standard solution**

**（1）Preparation of mixed standards:** Take 9920μL of n-butanol (HPLC grade), put it into a 15mL centrifuge tube, add 10μL of each of the 8 short-chain fatty acid standards, vortex and mix well to obtain the mixed standard stock solution A of the 8 short-chain fatty acids.

**（2）Preparation of internal standard:** Take 9990μL of n-butanol (HPLC grade), put it into a 15mL centrifuge tube, add 10μL of internal standard 2-ethylbutyric acid, and vortex and mix well to obtain the internal standard stock solution B.

The concentration of the stock solution is shown in the following table.

Table 1 Short-chain fatty acid standard stock solution information

| Name | Abbreviation | Concentration(μg/mL) |
| --- | --- | --- |
| Aceticacid | Ace | 1000 |
| Propanoicacid | Pro | 1000 |
| Butanoicacid | But | 1000 |
| Isobutyric | acid Isobut | 1000 |
| Valericacid | Val | 1000 |
| Isovalericacid | Isoval | 1000 |
| Hexanoicacid | Hex | 1000 |
| Isohexanoicacid | Isohex | 1000 |
| 2-ethylbutyricacid | Ethbut | 1000 |

The above mixed standard A and B solutions were diluted with n-butanol into 7 different concentrations of working solutions (Table 2) and loaded into the injection vials for GC-MS analysis.

Table 2 Information on the gradient concentrations of short-chain fatty acid mixed standards (μg/mL)

| Abbreviation | 1 | 2 | 3 | 4 | 5 | 6 | 7 |
| --- | --- | --- | --- | --- | --- | --- | --- |
| Ace | 0.1 | 0.5 | 1 | 5 | 10 | 50 | 100 |
| Pro | 0.1 | 0.5 | 1 | 5 | 10 | 50 | 100 |
| But | 0.1 | 0.5 | 1 | 5 | 10 | 50 | 100 |
| Isobut | 0.1 | 0.5 | 1 | 5 | 10 | 50 | 100 |
| Val | 0.1 | 0.5 | 1 | 5 | 10 | 50 | 100 |
| Isoval | 0.1 | 0.5 | 1 | 5 | 10 | 50 | 100 |
| Hex | 0.1 | 0.5 | 1 | 5 | 10 | 50 | 100 |
| Isohex | 0.1 | 0.5 | 1 | 5 | 10 | 50 | 100 |
| Ethbut | 10 | 10 | 10 | 10 | 10 | 10 | 10 |

**2.2 Sample processing**

1. Weigh 25mg of stool sample in 2ml grinding tube and add 500μL of water (containing 0.5% phosphoric acid).

2. Freeze and grind the sample for 3 min (50HZ) twice, then sonicate for 10 min and centrifuge at 4 ℃ and 13000g for 15 min.

3. Remove all supernatant water solution to 1.5mL centrifuge tube, and then add 0.2mL n-butanol solvent (containing 10μg/mL of internal standard 2-ethylbutyric acid) to extract. 4.

4. Vortex for 10s, sonicate at low temperature for 10min, centrifuge at 4℃ and 13000g for 5min, and take the supernatant solution to the injection vial for machine.

**2.3 GC-MS detection**

The analytical instrument for this experiment was an AgilentTechnologies Inc. 8890B-5977B GC/MSD gas chromatograph.

Chromatographic conditions: HPFFAP capillary column (30m×0.25mm×0.25μm, AgilentJ&WScientific, Folsom, CA, USA), high purity helium (purity not less than 99.999%), flow rate of 1.0 mL/min, inlet temperature of 260℃. The injection volume was 1 μL, and the sample was injected in a split flow with a split ratio of 10:1 and a solvent delay of 2.5 min. The initial temperature of the column was 80°C, and the column was ramped up to 120°C at 40°C/min, 200°C at 10°C/min, and then 230°C for 3 min.

Chromatographic conditions: electron bombardment ion source (EI), ion source temperature 230°C, quadrupole temperature 150°C, transmission line temperature 230°C, electron energy 70 eV. The scanning mode was selected ion scanning mode (SIM).

**2.4 Data analysis**

The default parameters of Masshunter quantification software (Agilent, USA, version: v10.0.707.0) were used to automatically identify and integrate the target short The default parameters of Masshunter quantification software (Agilent, USA, v10.0.707 0) were used to automatically identify and integrate the ionic fragments of the target short-chain fatty acids, and to assist in manual inspection. The detection concentration of each sample is calculated from the standard curve. The actual content of short-chain fatty acids in the sample is converted from the standard curve to the measured concentration of each sample.

Table 3 Main instrument

| Name of device | Manufacturers | product model | Instrument number |
| --- | --- | --- | --- |
| Multi-sample freeze grinder | Wonbio | Wonbio-96c | MJPRO-YQ-104 |
| Ultrasonic constant temperature cleaning machine | SCIENTZ | SBL-10DT | MJPRO-YQ-106 |
| High speed refrigerated centrifuge | Eppendorf | Centrifuge5430R | MJPRO-YQ-016 |
| Manual single channel pipettes | Eppendorf | (100-1000)μL | MJPRO-YQ-050 |
| Manual single channel pipettes | Eppendorf | (20-200)μL | MJPRO-YQ-053 |
| Manual single channel pipettes | Eppendorf | (10-100)μL | MJPRO-YQ-046 |
| Manual single channel pipettes | Eppendorf | (2-20)μL | MJPRO-YQ-047 |
| Manual single channel pipettes | Eppendorf | (0.5-10)μL | MJPRO-YQ-061 |
| Analytical Balance | Zhuo Jing | BSM-220.4 | MJPRO-YQ-009 |
| NewClassicMS | METTLERTOLEDO | NewClassicMFMS105DU | MJPRO-YQ-096 |
| Gas chromatograph- mass spectrometer | Agilent | Agilent8890B-5977B | MJPRO-YQ-105 |

Table 4 Main reagents

| Reagents and consumables | Article number | purity | Manufacturer |
| --- | --- | --- | --- |
| Aceticacid | MKCF0674 | 99.70% | SIGMA |
| Propanoicacid | 40049461 | 99.50% | WOKAI |
| Butanoicacid | STBF8413V | 99.50% | SIGMA |
| Isobutyric | H1823082 | 99% | SIGMA |
| Valericacid | STBG4549V | 99% | SIGMA |
| Isovalericacid | BCBW4945 | 99% | SIGMA |
| Hexanoicacid | MKCC8342 | 99% | SIGMA |
| Isohexanoicacid | MKCH9032 | 99% | SIGMA |
| 2-ethylbutyricacid | STBH0913 | 99% | SIGMA |
| phosphate | 10015418 | — | HUSHI |
| HPLC | N-butyl alcohol | A383-4 | — |
