# Supplementary material for: Evaluation of two laboratory model methods for diarrheal irritable bowel syndrome
Source: Mol Med. 2023 Jan 12;29:5. doi: 10.1186/s10020-022-00599-x (PMC9837933; doi:10.1186/s10020-022-00599-x)
Supplement: Supplementary file 2 — Additional file 2. Extended figures of intestinal flora. [file 10020_2022_599_MOESM2_ESM.docx]

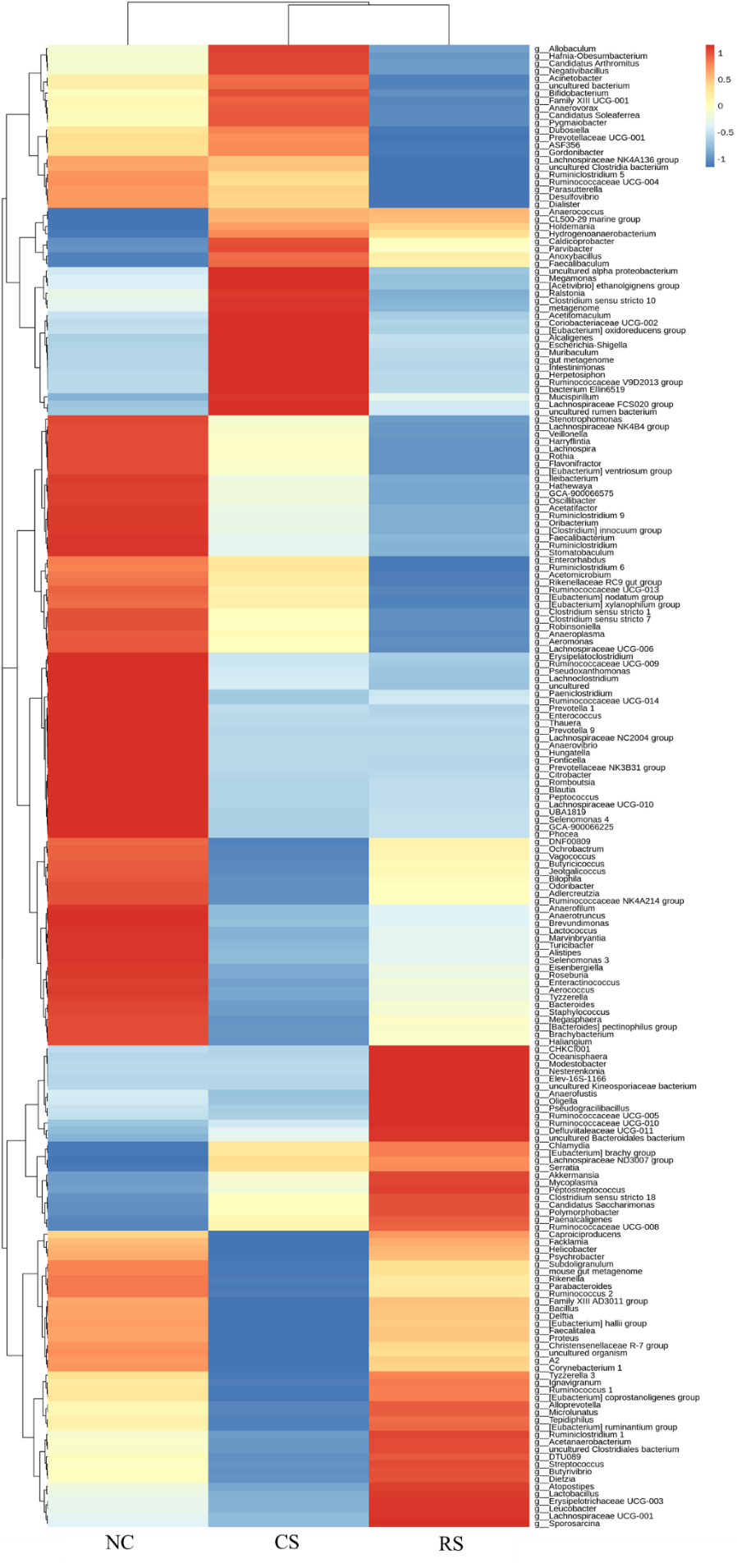


Figure8(A) Heat map analysis of species richness clustering at the genus level


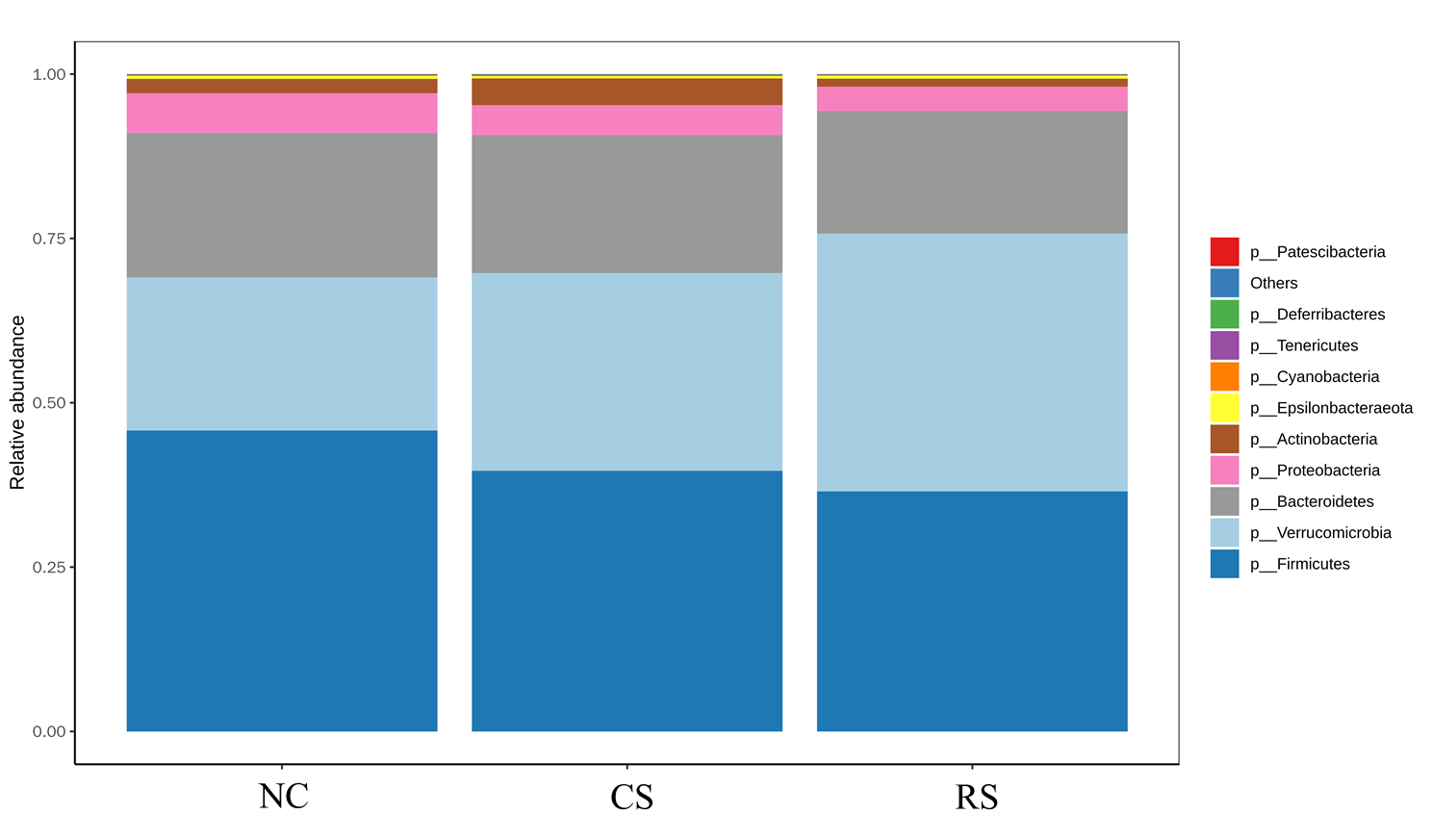


Figure8(B) Histogram of species richness at the phylum level for each group of samples


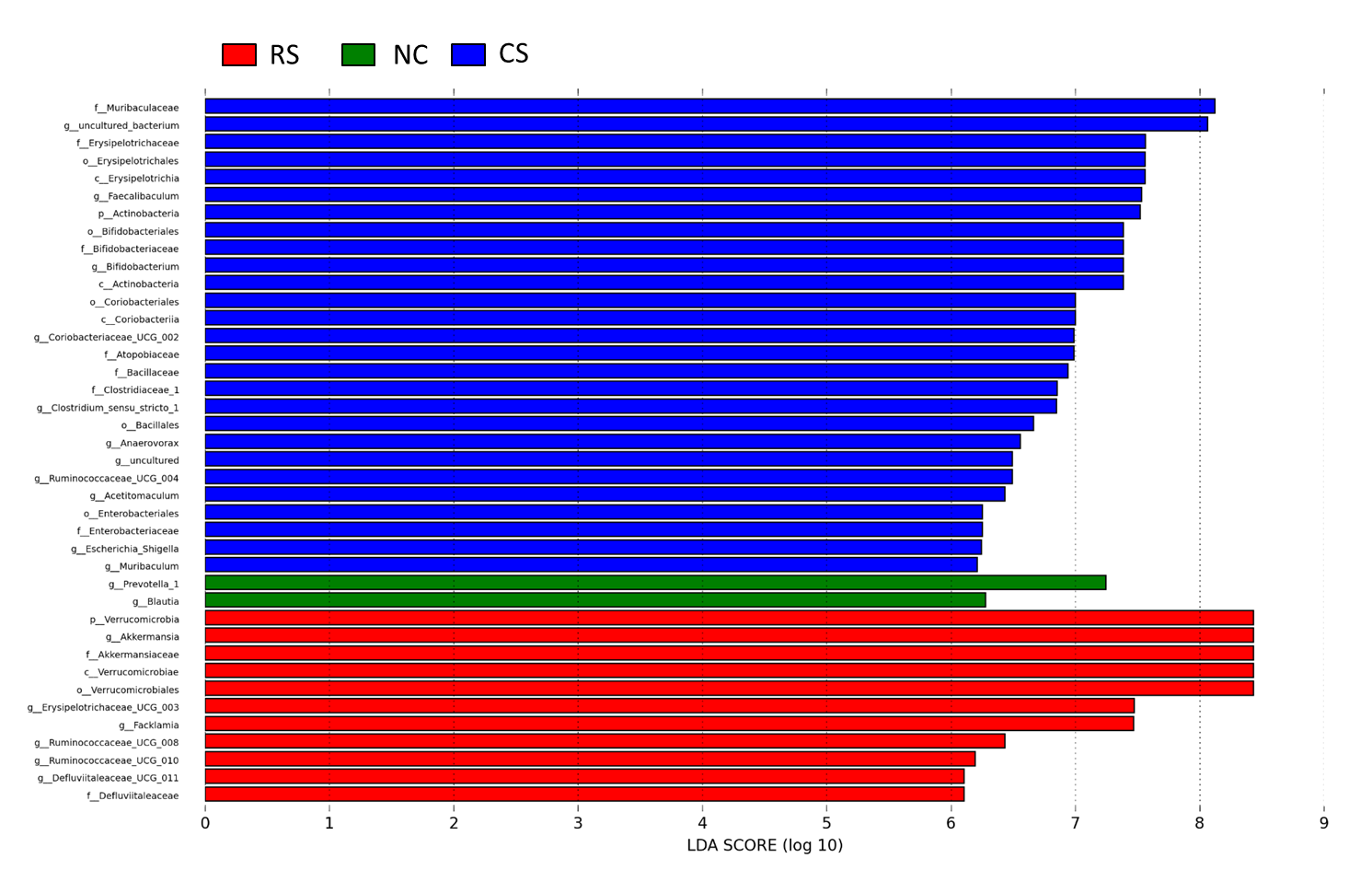


Figure8(C) Linear discriminant analysis(LDA) Score

Figure8(D)
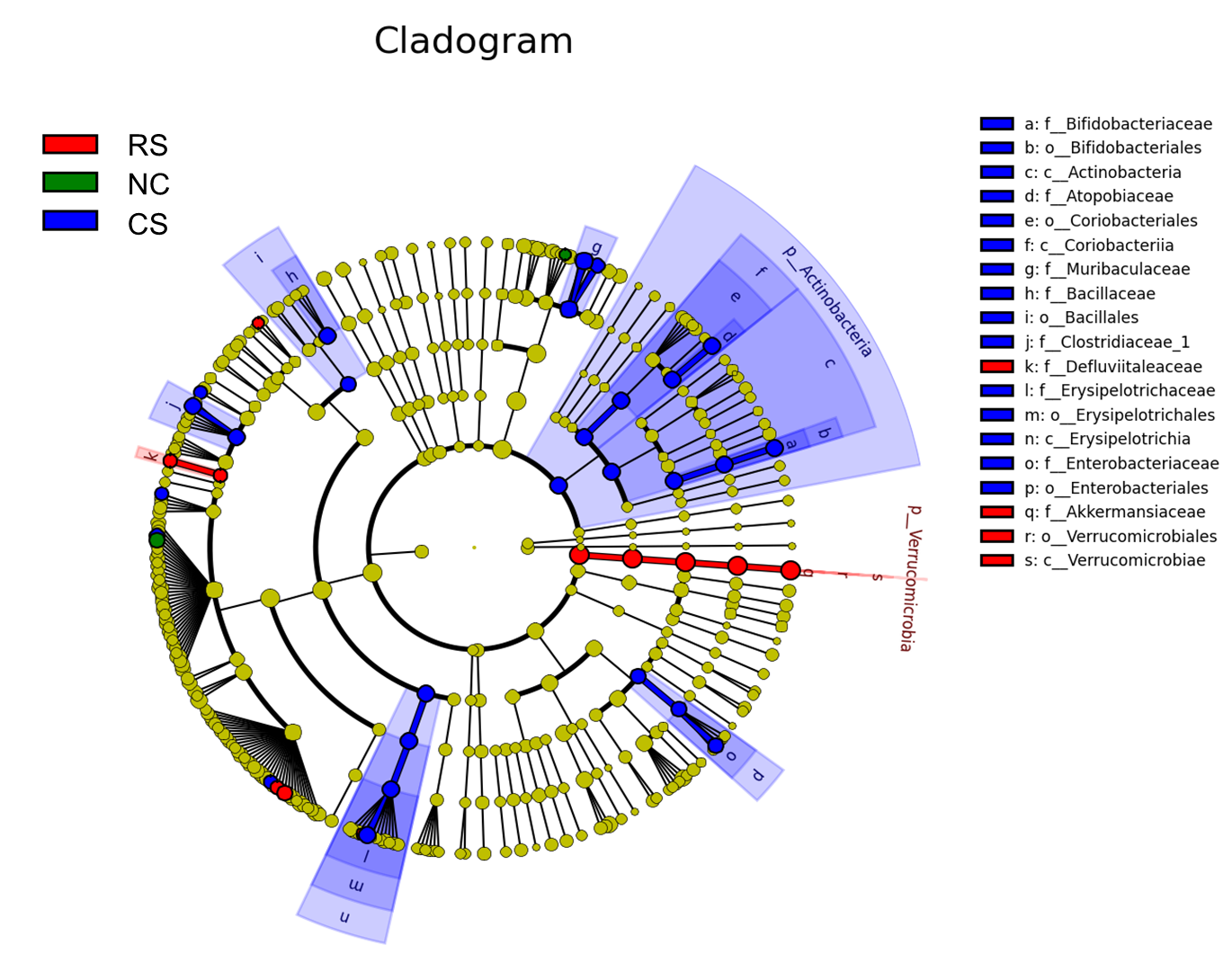
Evolutionary branching diagram
